# Parents' depression and loneliness during pregnancy and respiratory infections in the offspring: A prospective birth cohort study

**DOI:** 10.1371/journal.pone.0203650

**Published:** 2018-09-07

**Authors:** Linnea Schuez-Havupalo, Elina Lahti, Niina Junttila, Laura Toivonen, Minna Aromaa, Päivi Rautava, Ville Peltola, Hannele Räihä

**Affiliations:** 1 Department of Pediatrics and Adolescent Medicine, Turku University Hospital and University of Turku, Turku, Finland; 2 Outpatient Clinic for Children and Adolescents, City of Turku, Turku, Finland; 3 Department of Psychology, University of Turku, Turku, Finland; 4 Department of Teacher Education, University of Turku, Turku, Finland; 5 Department of Public Health, University of Turku and Clinical Research Centre, Turku University Hospital, Turku, Finland; Chiba Daigaku, JAPAN

## Abstract

**Background:**

An association between maternal prenatal stress and increased rates of respiratory tract infections in the offspring has been described earlier. Data regarding the father’s role is lacking. In this study our aim was to evaluate, whether mothers’ and fathers’ depressive symptoms and loneliness during pregnancy predict higher rates of respiratory tract infections in the offspring.

**Methods:**

In this longitudinal cohort study we gathered information on parental psychological risk during gestational week 20 using the BDI-II and UCLA loneliness scale questionnaires for the parents of 929 children. Loneliness was divided into social and emotional components, the former describing patterns of social isolation and the latter a perceived lack of intimate attachments. Episodes of acute otitis media, physician visits due to respiratory tract infections, and antibiotic consumption relating to respiratory tract infections were documented in the infants, excluding twins, from birth until 10 months of age using study diaries. Analyses were carried out by structural equation modeling, which provides dynamic estimates of covariances.

**Results:**

Maternal depressive symptoms during pregnancy predicted higher rates of acute otitis media in the infant and maternal emotional loneliness predicted higher rates of physician visits. Acute otitis media, physician visits and antibiotic consumption in the infant were slightly less frequent for families who reported social loneliness in the father or mother. Associations remained when taking into account confounders.

**Conclusions:**

Maternal prenatal depression and emotional loneliness predicted a higher burden of respiratory tract infections in the offspring. The protective influence of parental social loneliness on the burden of respiratory tract infections in infants was not in line with our study hypothesis, but could be explained by reduced use of healthcare services in these socially isolated families.

## Introduction

Respiratory tract infections (RTIs) are the most common reason for medical care and antibiotic prescriptions in children [1]. Given the substantial disease burden to children, families, and society [1], new insights in how to reduce childhood RTIs are needed.

Identified risk factors for childhood RTIs include age, gender, number of siblings, day care attendance, exposure to smoking, genetic factors, socioeconomic status, and parents’ long-term illnesses [2,3]. Parental psychological factors, such as maternal stress during pregnancy, can also have an influence on the burden of infectious diseases in the offspring [4–6]. In the Norwegian Mother and Child Cohort Study, prenatal stressful life events, as well as relationship dissatisfaction, were associated with a higher frequency of infectious diseases in the infants [7]. To our knowledge, only one previous study has investigated the association between prenatal depression and the risk of childhood respiratory infections [8]. In this population-based cohort study conducted in the United Kingdom, children of mothers with perinatal depression were found to have a 27% increased risk of lower RTIs compared to children of mothers without perinatal depression, and the rates of overall childhood infections were consistently higher throughout the first years of life. There are no studies on the role of paternal depression.

The impact of loneliness on health is a relatively new research area. Loneliness has been linked with mental and physical health problems in adults [9,10], and with alterations in healthcare-seeking behavior [11,12]. The role of parental loneliness regarding the rates of RTIs in children has not been studied. We consider parental loneliness a valuable measure of psychological risk, since it strongly correlates with parental anxiety, depression, and low self-efficacy [13], all of which are considered important determinants with regard to psychological problems in the child [14,15]. Compared to depression, parental loneliness may be less subject to denial in a community setting. Two distinct types of loneliness can be recognized; social loneliness, which is characterized by social isolation, and emotional loneliness, characterized by a lack of intimate attachments.

Within the context of a large Finnish cohort study (the Steps study), we aimed to examine, whether mothers’ and fathers’ antenatal depressive symptoms and loneliness are associated with higher rates of acute otitis media (AOM), RTI-related physician visits and antibiotic treatments in infants, and whether they have an influence on parental healthcare-seeking behavior. We hypothesized that parental depression and loneliness during pregnancy predict a higher burden of RTIs in the offspring.

## Materials and methods

### Study population and conduct

**Fig 1:**
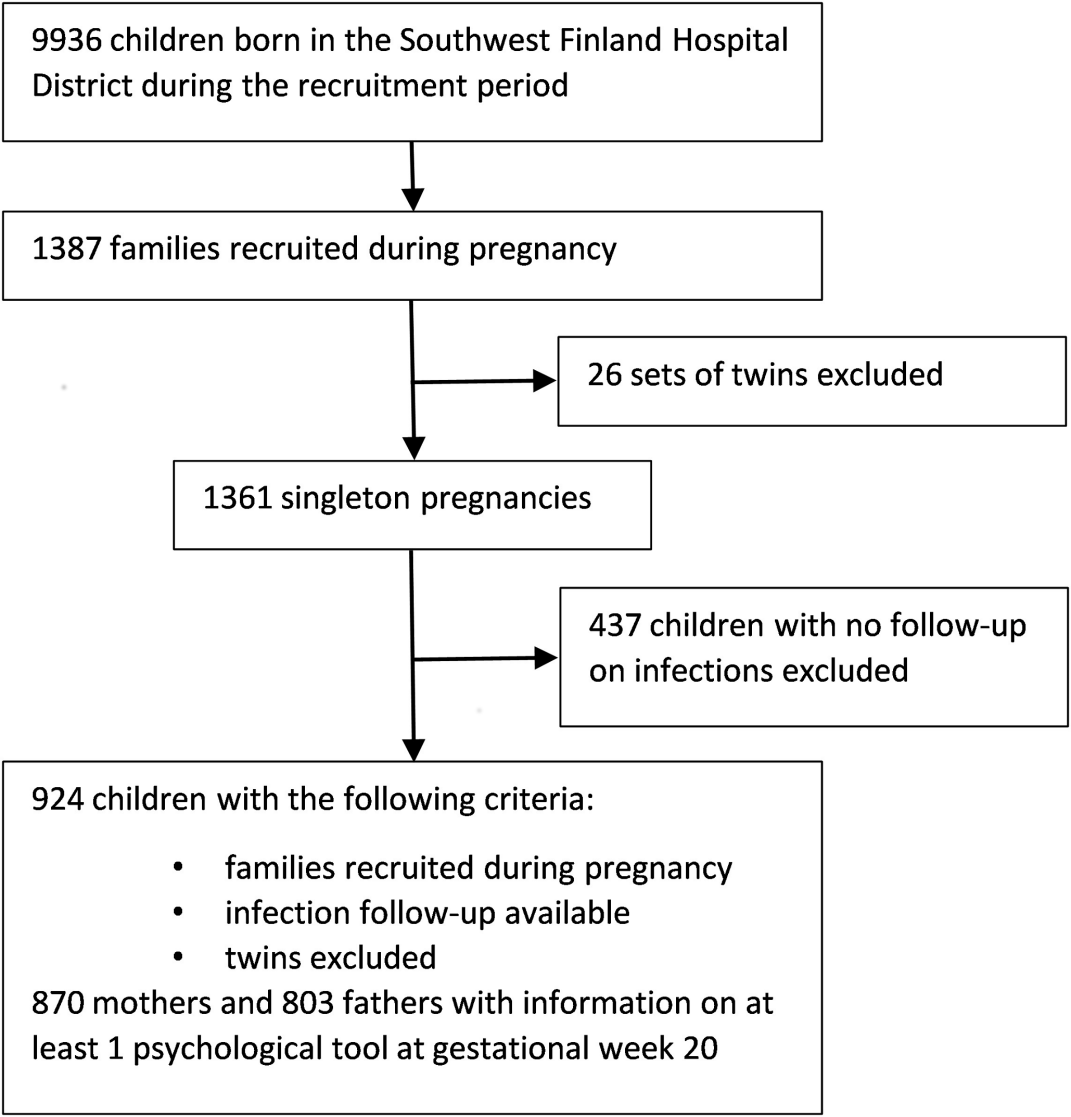
Flow-chart showing the recruitment to our study.

We used the birth cohort of the observational ‘Steps to the Healthy Development and Well-being of Children’ Study, which includes registry data from all 9936 children born between 1 January 2008 and 31 March 2010 in the Southwest Finland Hospital District. The Steps study is a multi-professional population-based study designed to assess various aspects of child development and health [16]. Altogether, 1797 families (1827 children) consented to be part of the Steps study during pregnancy or shortly after birth. Of those, 1387 families were recruited during pregnancy between the 10^th^ and 15^th^ gestational week during routine visits to community midwifery services. The 410 families who joined the study after the birth of their child had no available information on psychological measures during pregnancy, and could therefore not be included in the follow-up described here. After exclusion of 26 sets of twins and 437 children with no follow-up on infections, there were 924 children within the active follow-up (Fig 1).

During gestational week 20 we collected questionnaire data on family-related factors and assessed parental loneliness and depressive symptoms using the tools specified below. Children were followed up prospectively by detailed diaries where parents documented day-to-day information on symptoms, physician visits, antibiotic medications and other factors. The symptom check-lists in the diaries contained also a record of symptom-free days in order to differentiate negative from missing information. Part of the cohort (n = 566 children) were invited to visit our physician-led study clinic during RTIs, thereby enabling us to obtain direct clinical information from these children. Participation in this sub-cohort was offered to all families without any exclusion criteria.

The Ethics Committee of the Hospital District of Southwest Finland approved the study. The parents of the participating children gave their written, informed consent.

### Psychological measures

To assess the parents´ depressive symptoms, Beck’s Depression Inventory (BDI-II) [17] was adopted. The Finnish version of BDI-II includes 21 items with four choices (e.g. 0 = I don´t feel disappointed in myself / 1 = I am disappointed in myself / 2 = I am disgusted with myself / 3 = I hate myself).

To evaluate the parents’ social and emotional loneliness during pregnancy, we used the Finnish version of the UCLA loneliness scale [18,19]. The scale includes factors of social loneliness (e.g. “I feel left out”, “I am unhappy being so withdrawn”) and emotional loneliness (e.g. “No one really knows me well”, “I am no longer close to anyone”) rated on a 4-point scale (1 = never; 2 = rarely, 3 = sometimes; 4 = often). Loneliness is subjective anxiety causing feelings of being without the type of relationships the person desires, i.e. a discrepancy between one's real and desired relationships. Social loneliness refers to the absence of a social network, or to the feeling that one is not part of a group. Emotional loneliness, in turn, refers to the lack of a close, intimate attachment to another person [18,20].

### Outcome measures

The outcome measures of the study were episodes of AOM, number of physician visits due to RTIs, and antibiotic medications due to RTIs from birth to 10 months of age. AOM was defined as acute infection of the middle ear with documented clinical signs and symptoms of infection. Regarding repeated diagnoses of AOM during continuous symptoms, an interval of 14 days was required before a new diagnosis was accepted.

### Confounding factors

The following potential confounders were taken into consideration: parental age, parental post-secondary education (stratified into eight groups with ascending educational levels), parental chronic illnesses (including psychiatric diagnoses, such as depression), breast feeding (exclusive or partial breast feeding until the age of 6 months), gender of the infant, older siblings in the family, and preterm birth before 37 weeks of gestation.

### Statistical analysis

**Fig 2:**
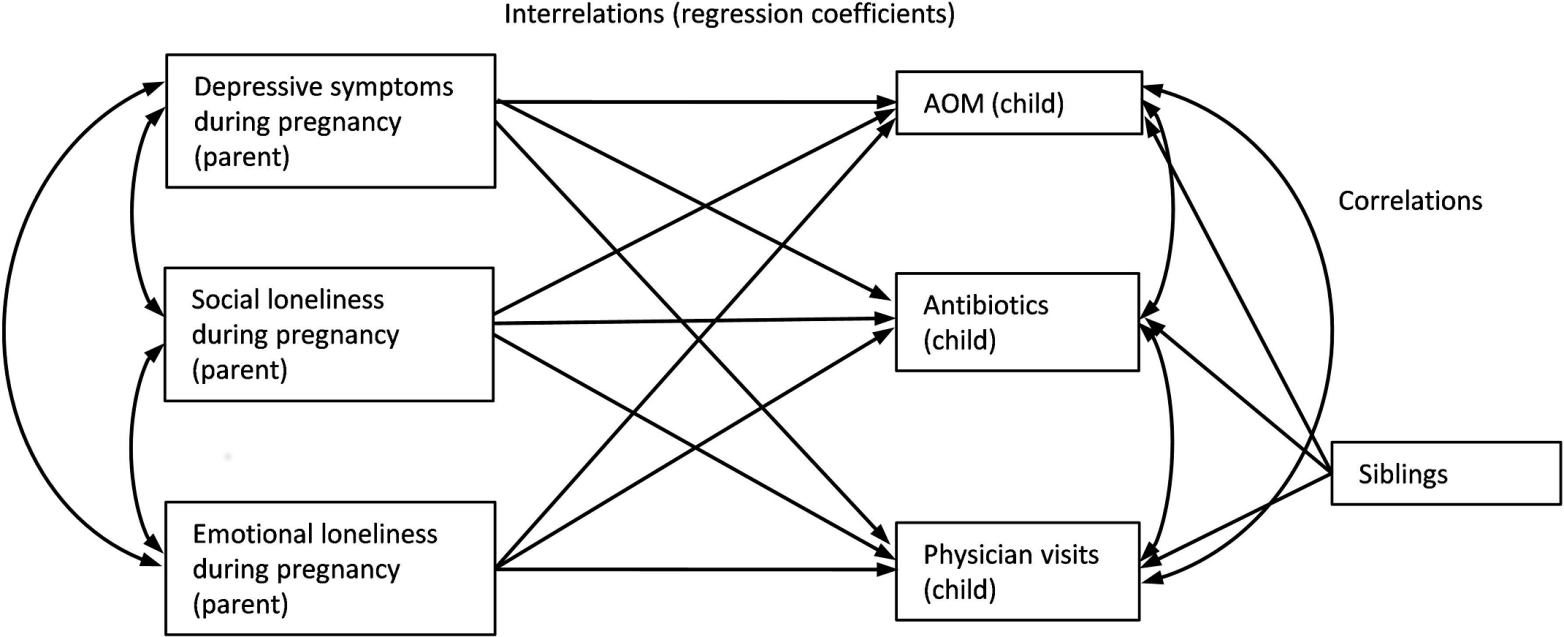
Hypothesized model.

We used structural equation modeling (SEM), which constitutes a second generation multivariate technique allowing for simultaneous processing of several dependent variables, thereby obtaining a dynamic estimate of covariances and correlations between variables. The analyses were conducted using Mplus 7.3 (Muthén and Muthén, Los Angeles, CA) [21] and the fit of the models was evaluated using a chi-square, the root mean square error of approximation (RMSEA), a comparative fit index (CFI), the Tucker-Lewis Index (TLI) [22], and the standardized root mean square residual (SRMR) [23–25]. The overall fit of models was considered to be acceptable with the following criteria being fulfilled: chi-square P values over 0.05, or chi-square values of more than three times the degree of freedom (DF), CFI and TLI of 0.90 or higher, and RMSEA and SMSR values below 0.08. The effects of mothers´ and fathers´ depressive symptoms and social and emotional loneliness during pregnancy on their child´s risk of AOM, antibiotic medications, and physician visits were analyzed using path modeling, a type of SEM. The hypothesized model is shown in Fig 2. Similar analyses were carried out for the effect of background variables on the child’s risk of AOM, antibiotic medications, and physician visits. Effect sizes were measured by standardized regression coefficients. Cohen’s d, which is the difference between two means divided by a standard deviation of the data, was used while evaluating the differences between mothers’ and fathers’ background variables. The reliability of psychological measures was assessed using Crohnbach’s alpha [18]. The estimates were 0.79 for mothers´ and 0.77. for fathers´ social loneliness; 0.77 for mothers´ and 0.78 for fathers´ emotional loneliness and 0.83 for mothers´ and 0.84 for fathers´ depressive symptoms.

## Results

Comparing mothers and fathers, mothers had more depressive feelings (Cohen’s d 0.948) and fathers more emotional loneliness (Cohen’s d 0.282) at gestational week 20 (Table 1). Among background variables (Table 2), older siblings in the family, fathers’ age and mothers’ educational levels had significant effects on the rates of AOM, physician visits, and antibiotic consumption due to RTIs in the child. Since the effect of older siblings was significant on all three outcome variables (standardized regression coefficients for AOM, 0.260, antibiotic medication, 0.281, and for physician visits, 0.191) we controlled its effect in the final models.

**Table 1:** Descriptive results, reliability coefficients (Cronbach’s alpha) and differences between mothers and fathers.

					Differences between mothers (M) and fathers (F)	
	Min. / max.	Mean (sd)	Skewness / kurtosis	Cronbach´s alpha	p-value	Cohen´s d
Mothers (at gestational week 20)						
Age, years	17 / 43	30.67 (4.32)	0.04 / 0.08			
Depressive symptoms, score	0 / 49	8.76 (6.01)	1.66 / 5.28	.83		
Social loneliness, score	6 / 21	9.63 (2.67)	1.14 / 1.33	.79		
Emotional loneliness, score	6 / 21	8.78 (2.30)	1.71 / 4.04	.77		
Fathers (at mother’s gestational week 20)						
Age, years	18 / 56	32.67 (5.29)	0.62 /1.01		<0.001	.414 M < F
Depressive symptoms, score	0 / 30	3.66 (4.66)	2.01 / 5.13	.84	<0.001	.948 M > F
Social loneliness, score	6 / 20	9.60 (2.67)	1.06 / 1.11	.77	0.68	ND
Emotional loneliness, score	6 / 21	9.47 (2.58)	1.21 / 1.66	.78	<0.001	.282 M < F
Children						
Acute otitis media, No. / 0–10 months of age	0 / 6	0.56 (0.96)	2.06 / 4.46			
Antibiotics, No. / 0–10 months of age	0 / 9	0.74 (1.25)	2.38 /7.22			
Physician visits for RTI, No. / 0–10 months of age	0 / 14	1.74 (2.17)	1.78/ 3.82			

F, Fathers; M, mothers; ND, not defined; SD, standard deviation

**Table 2:** Associations of background variables with outcome measures.

		Standardized Regression Coefficient (P)		
	N (%)/ group total	AOM	Antibiotics	Physician visits
Child’s gender (boys)	461 (50)/ 924	0.056 (0.10)	0.034 (0.34)	0.056 (0.11)
Older siblings	448 (48)/ 924	**0.260 (<0.001)**	**0.281 (<0.001)**	**0.191 (<0.001)**
Born preterm (< 37 th gestational week)	34 (4)/ 913	-0.003 (0.91)	-0.009 (0.74)	-0.012 (0.66)
Breastfeeding (until 6 months of age)	503 (61)/ 831	0.005 (0.88)	0.007 (0.84)	0.001 (0.99)
Chronic illness/ father	367 (42)/ 873	-0.059 (0.09)	-0.059 (0.09)	-0.029 (0.43)
Chronic illness/ mother	456 (51)/ 895	0.025 (0.49)	0.056 (0.11)	0.039 (0.29)
Fathers’ age	-/ 924	-0.071 (0.09)	**-0.103 (0.01)**	**-0.130 (0.003)**
Mothers’ age	-/ 924	0.022 (0.62)	0.023 (0.59)	0.024 (0.59)
Mothers’ education, lower (lower 4 groups)	336 (37)/ 912	**-0.092 (0.01)**	-0.040 (0.29)	-0.039 (0.31)
Fathers’ education, lower (lower 4 groups)	477 (54)/ 887	0.007 (0.84)	0.018 (0.60)	0.016 (0.70)

Statistically significant values are bolded. Children’s outcome measures were documented during 0–10 months of age. The variables of parental age and post-secondary education (8 groups) were analyzed as continuous variables.

N = 749.

**Fig 3:**
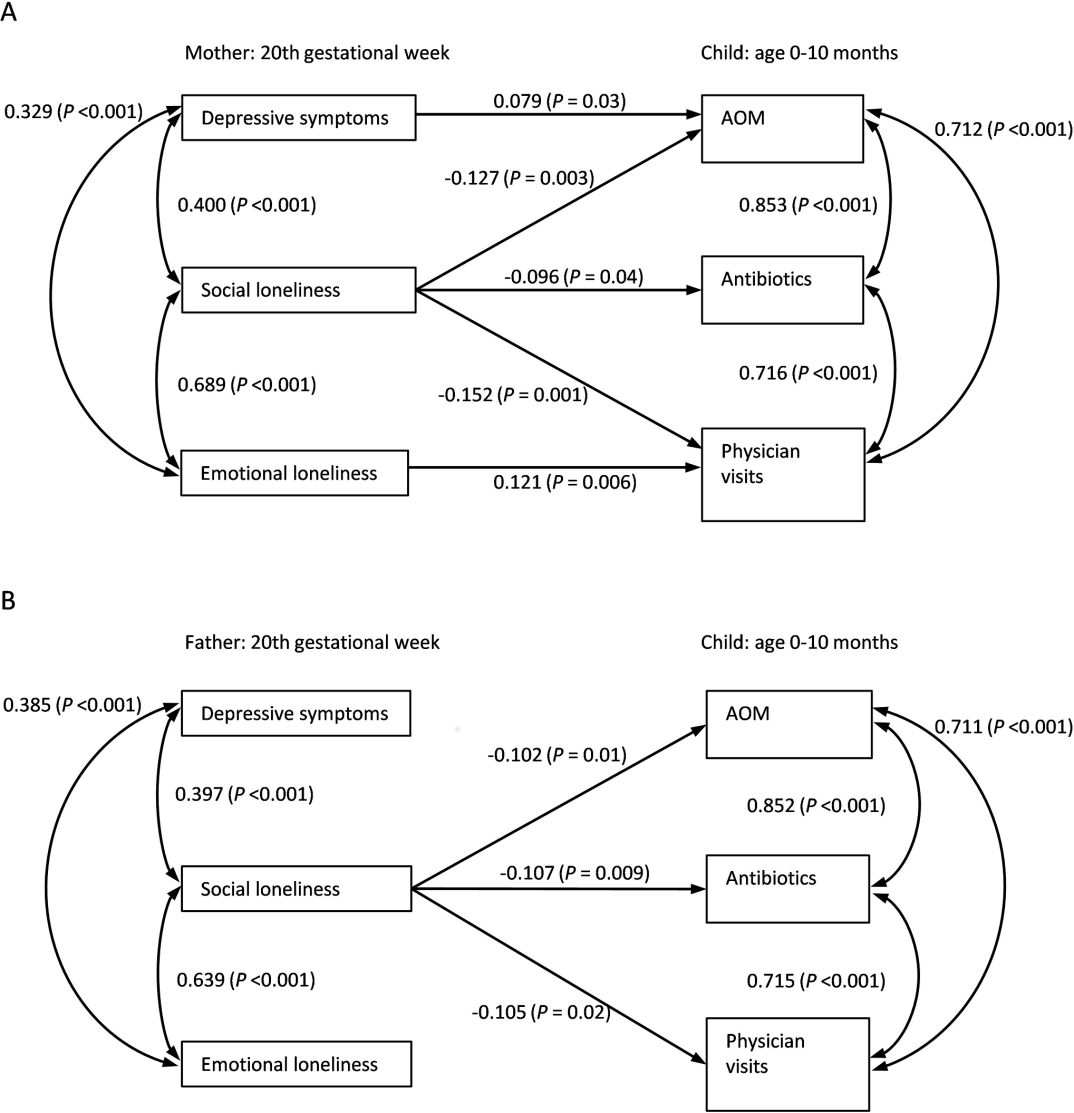
Mothers’ (panel A) and fathers’ (panel B) loneliness and depressive symptoms during pregnancy predicting acute otitis media (AOM), antibiotics, and physician visits in the offspring. Standardized regression coefficients and P values after controlling the effect of siblings. The standardized regression coefficients for the effect of siblings were 0.235 and 0.243 for AOM, 0.252 and 0.258 for antibiotics, and 0.162 and 0.172 for physician visits in the analyses of mothers’ and fathers’ psychological factors, respectively.

The effects of mothers´ and fathers´ depressive symptoms and social and emotional loneliness during pregnancy on their child´s risk of AOM, antibiotic medications, and physician visits until the age of 10 months were analyzed using path modeling. The resultant models are presented in Fig 3 (panel A for mothers, panel B for fathers). The goodness of fit indices estimated at least acceptable fit for both models (for mothers: chi-square, 23.158 with DF, 3; CFI = 0.974; TLI = 0.869; RMSEA = 0.085; SRMR = 0.039; for fathers: chi-square, 3.906 with DF, 3 and chi-square P, 0.27; CFI = 0.999; TLI = 0.994; RMSEA = 0.018; SRMR = 0.018). The extremely good fit for fathers supported the hypothesized model, in spite of less favorable fit indices for mothers.

Depressive symptoms (Fig 3, panel A) during pregnancy in mothers predicted higher numbers of AOM in their infant during the follow-up (P = 0.03). The effect of fathers’ depressive symptoms on children’s rates of AOM, antibiotic medications, and physician visits was below the level of statistical significance. Feelings of emotional loneliness in the mother predicted more physician visits for the child (P = 0.006). There were no corresponding significant findings for effects of emotional loneliness in the father. The effect of social loneliness was contradictory to others, i.e. more feelings of social loneliness predicted lesser numbers of AOM (P = 0.003/ mother, P = 0.01/ father), antibiotic consumption (P = 0.04/ mother, P = 0.009/ father), and physician visits (P = 0.001/ mother, P = 0.02/ father).

## Discussion

Our study has two important findings. First, we found an association between mothers’ depressive symptoms during pregnancy and an increased frequency of AOM diagnoses in infants. Second, parental loneliness during pregnancy was associated with numbers of AOM diagnoses, antibiotic medications, and physician visits in infants. Maternal emotional loneliness was associated with increased physician visits, but parental social loneliness resulted in slightly decreased numbers of AOM diagnoses, antibiotic medications, and physician visits. To our knowledge, this is the first report of this kind, describing an interrelationship between parental prenatal loneliness and infant outcomes.

The possible mechanisms behind the effects of parental prenatal depressive symptoms and loneliness on outcomes in the child are numerous. It has been suggested that maternal psychological factors during pregnancy have programming effects on the function of the hypothalamic-pituitary-adrenocortical axis in the offspring [26,27], and may thereby influence the developing immune system with regard to immune programming and subsequent disease susceptibility [28–30]. In line with this hypothesis, recent studies have reported an association between maternal prenatal stress, anxiety and depression, and an increase in infectious diseases in infants [4,5,7,8]. Another possible mechanism is that parental prenatal depression and loneliness can have an impact on healthcare-seeking behavior in families, as those factors tend to be relatively stable from pregnancy to the postnatal period in both parents [18,31]. Previously, maternal depression has been found to be associated with an increased use of acute healthcare for infants [32], and maternal prenatal loneliness with increased unscheduled hospital visits during pregnancy [11]. Other possible mechanisms to explain the association between maternal psychological factors and infectious diseases in infants include mothers’ poor life style choices and poor nutritional status during pregnancy that may be linked to depression [33]. An interesting new hypothesis is the influence of maternal gut microbiota on the development of the infants’ immune system [34].

In our study, depressive symptoms in mothers were significantly associated with an increase in numbers of documented AOM, but not with an increase in numbers of physician visits, suggesting that mothers’ prenatal depression may increase true infection-proneness in infants. Fathers’ prenatal depression, which naturally lacks the direct programming effect on the child, did not have corresponding influence on the child. The effect of loneliness on child outcome varied depending on its type. Maternal emotional loneliness predicted higher rates of physician visits in infants, but parental social loneliness predicted reduced rates of physician visits, AOM, and antibiotic medications in infants. This bidirectional influence of parental loneliness could be explained by different patterns in healthcare-seeking behavior. Emotional loneliness may increase the mother’s need for outside support and guidance, and, taking into account that the decision to visit a doctor may often be made by the mother, this may lead to recurrent physician visits with an infant. Social loneliness, in turn, may cause avoidant-type behavior with respect to social tasks, such as medical consultations, leading to decreased numbers of physician visits and thereby resulting in decreased numbers of otitis media diagnoses and antibiotic prescriptions. Social loneliness in parents may also influence the frequency of social activities and may thereby reduce exposure to pathogens causing RTIs.

Previously, loneliness has been found to have an effect on healthcare-seeking behavior in adults [11]. There is also accumulating evidence that loneliness has broad-ranging negative effects on health including immune functioning [9]. Few studies have analyzed the influence of social support on healthcare-seeking behavior in families, and the results have been controversial. In a study by Riley et al, greater parental satisfaction with social support correlated with lower use of pediatric healthcare services [35]. On the contrary, in a study by Horowitz et al, parents from families with large social networks belonging to the middle- and upper-class were more likely to repeatedly use acute pediatric care services [36]. This may have been related to better access to healthcare and thereby low-threshold consultations as a result of superior resources. More data is needed on the influence of parental prenatal loneliness (both emotional and social dimensions) and social support on healthcare-seeking behavior in families and on the burden of RTIs in infants. As loneliness may be a more sensitive indicator of deficient psychological well-being compared to depressive symptoms in families [10], and as it is probably more easily admitted to health care professionals than depression, loneliness might be an effective screening method in order to assess psychological risk in families.

There is a growing body of evidence relating to the importance of fathers with respect to the child’s psychological development [37]. The paternal role expressed by marital quality has been described to have an impact on the frequency of RTIs in the offspring within the first year of life [7], and paternal need for outside support has been linked to recurrent use of antibiotics in children [2]. Findings in our study support the importance of the paternal role with regard to healthcare-seeking behavior in the family. Direct effects on infections in the offspring are less clear. In our analyses with background variables, there was a protective effect of higher paternal age with regard to physician visits and numbers of antibiotic medications. Older fathers might be able to provide stronger support to mothers, which in turn reduces the family’s need to seek for outside support and guidance.

Strengths and limitations to our study need to be reflected. We consider the main strength the detailed, prospective follow-up in a relatively large cohort in combination with a novel set of psychological measures. Our study design using path modeling enabled us to obtain a more precise insight into possible causalities in comparison with more traditional methods. Effect sizes observed were small, but statistically significant. Similar to other prospective cohort studies, the major limitation of this study is a potential selection bias with regard to the families who joined our study. Missing information in diaries and questionnaires of participating families was clearly differentiated from negative information, but it still may have further added some bias [38]. Background variables between the entire eligible cohort and families participating in the Steps study were compared around recruitment and at the children’s age of 13 months. These analyses revealed that maternal education and occupational income was slightly higher, and older siblings were less frequent in the study families compared to all eligible families [39]. Other minor differences constituted a slightly higher age in participating mothers and more likely urban residence in the corresponding families. Participating mothers were also more likely married [16]. Otherwise our cohort represents the Southwest Finnish population well.

We aimed to control for background variables as well as possible. This led to a relatively short follow-up period of 10 months of age in the infants, since in Finland out-of-home daycare is uncommon during this time interval [40], which is covered by parental leave. There were only 9 children who were known to commence out-of-home daycare by the age of 9 months and 33 children by the age of 10 months (22 in family daycare and 11 in daycare centres). Given the fact that in our cohort a daycare-related rise of RTIs seemed to be strongly dependent on chronological aspects [40], and since this rise was clearly smaller for children in family daycare than for those cared for in daycare centres, adjustment for daycare seemed neither feasible nor necessary. In Finland, prevalence of maternal smoking is relatively low at 15% during early pregnancy, and it tends to be associated with lower socioeconomic status [41]. Accordingly, in our study there was only a small proportion of children with a known exposure to parental smoking (146 out of 863 children with information on parental smoking). There were several indicators that diary follow-up in families with a smoking parent had not been completed as carefully as in the remainder of the cohort, and we were therefore not able to reliably assess this variable. Previous research has shown that smoking parents may underutilize health care services with respect to RTI symptoms in their children [42], and it is thus conceivable that this same tendency was also observable in our study.

The psychological data used in this study was based on self-report, but all psychological measures were validated. It is of note that we studied the subjective experience of depressive symptoms and loneliness, and not clinical diagnoses of depression or other mental disease. While this helped us to assess a real-life situation via a community-based approach, this research design may partially explain the small effect sizes observed. Chronic disease in the parents was considered as a background measure, and psychiatric diagnoses such as depression were included in this variable.

## Conclusions

Our study results strengthen the current understanding of the importance of parental psychological factors with regard to child well-being and health. Our findings suggest that a part of the burden of childhood RTIs can be explained by maternal prenatal depressive symptoms and emotional loneliness. In addition to postnatal depression, also prenatal depression and loneliness should gain attention in preventative medicine. Health care professionals treating infants with recurrent RTIs should take parental psychological factors into consideration and arrange psychological support when needed.

## Abbreviations

AOM: acute otitis media
CFI: comparative fit index
CI: confidence interval
DF: degree of freedom
RMSEA: root mean square error of approximation
RTI: respiratory tract infection
SD: standard deviation
SEM: structural equation model
SRMR: standardized root mean square residual
STEPS: Steps to the Healthy Development and Well-being of Children
TLI: Tucker-Lewis index
